# The second pregnancy has no effect in the incidence of macrosomia: a cross-sectional survey in two western Chinese regions

**DOI:** 10.1186/s41043-021-00244-z

**Published:** 2021-04-13

**Authors:** Li Luo, Huan Zeng, Mao Zeng, Xueqing Liu, Xianglong Xu, Lianlian Wang, Yong Zhao

**Affiliations:** 1 School of Public Health and Management, Chongqing Medical University, Chongqing, 400016 China; 2 Research Center for Medicine and Social Development, Chongqing Medical University, Chongqing, 400016 China; 3 Collaborative Innovation Center of Social Risks Governance in Health, Chongqing Medical University, Chongqing, 400016 China; 4 Chengdu Center for Disease Control and Prevention, 610041 Chengdu, China; 5 Central Clinical School, Faculty of Medicine, Nursing and Health Sciences, Monash University, 3800 Melbourne, Australia; 6 The Department of Obstetrics, The First Affiliated Hospital of Chongqing Medical University, 400016 Chongqing, China; 7 Department of Reproduction Health and Infertility, The First Affiliated Hospital of Chongqing Medical University, 400016 Chongqing, China; 8 Canada-China-New Zealand Joint Laboratory of Maternal and Fetal Medicine, Chongqing Medical University, 400016 Chongqing, China

**Keywords:** Fetal macrosomia, China, Parity, Cross-sectional study, Incidence

## Abstract

**Background:**

After the implementation of the universal two-child policy in China, the increase in parity has led to an increase in adverse pregnancy outcomes. The impact of one and two fetuses on the incidence of fetal macrosomia has not been fully confirmed in China. This study aimed to explore the differences in the incidence of fetal macrosomia in first and second pregnancies in Western China after the implementation of the universal two-child policy.

**Methods:**

A total of 1598 pregnant women from three hospitals were investigated by means of a cross-sectional study from August 2017 to January 2018. Participants were recruited by convenience and divided into first and second pregnancy groups. These groups included 1094 primiparas and 504 women giving birth to their second child. Univariate and multivariate logistic regression analyses were performed to discuss the differences in the incidence of fetal macrosomia in first and second pregnancies.

**Results:**

No significant difference was found in the incidence of macrosomia in the first pregnancy group (7.2%) and the second pregnancy group (7.1%). In the second-time pregnant mothers, no significant association was found between the macrosomia of the second child (5.5%) and that of the first child (4.7%). The multivariate logistic regression model showed that mothers older than 30 years are not likely to give birth to children with macrosomia (odds ratio (OR) 0.6, 95% confidence interval (CI) 0.4,0.9).

**Conclusions:**

The incidence of macrosomia in Western China is might not be affected by the birth of the second child and is not increased by low parity.

## Background

Fetal macrosomia is a term used to define a newborn that is significantly larger than average, with a birth weight ≥ 4000 g or in the 90th percentile in terms of gestational age [1]. In recent years, the incidence of fetal macrosomia has increased globally [2–4]. Fetal macrosomia is suggested to be associated with increased risk of adverse outcomes in the mother and the infant [5]. Some previous studies showed that the most important neonatal complications of macrosomia are shoulder dystocia, perinatal asphyxia, birth injury, hypoglycemia, meconium aspiration syndrome, and death [5–7]. Additionally, fetal macrosomia is likely to lead to obesity, hypertension, and type-2 diabetes mellitus in adulthood [8]. Fetal macrosomia increases the risk of postpartum hemorrhage, infection, cesarean delivery, prolonged labor, high-degree perineal tears, anesthetic accidents, and thromboembolism [9]. In 2013, the Chinese government introduced a policy that allows couples, at least one of which is an only child, to have two children; in October 2015, China implemented the universal two-child policy [10]. With a large number of families eager to have two births, the birth peak of two children has come under the influence of national policies, leading to an increase in parity and the consequent increase in pregnancy risks. With the improvement of the living standards of Chinese people, the incidence of fetal macrosomia increased from 6% in 1995 to 7.8% in 2005 [11, 12]. Multiple surveys conducted in various regions of China showed an increase in the incidence of fetal macrosomia. For example, a survey conducted in Harbin, China, showed that the incidence of fetal macrosomia increased from 8.31% in 2001 to 10.50% in 2005 [13]. Similarly survey data from Shanghai revealed a 50% increase in the incidence of fetal macrosomia from 1989 to 1999 [14]. Survey data collected across 14 provinces in China showed a mean macrosomia incidence of 7.3%, ranging from 4.1 to 13.4% [15].

The causes of fetal macrosomia are complex and mostly remain unexplained. Some risk factors have been identified, and these factors include high pre-pregnancy body mass index (BMI), excessive weight gain during pregnancy, prolonged gestation, male fetal sex, multiparity, maternal age, maternal height, pre-gestational diabetes, and gestational diabetes [16–18]. Among them, parity is one of the important factors affecting the occurrence of fetal macrosomia. Studies showed that the maternal peritoneal and uterine wall of multiparas is more relaxed than that of primiparas. Changes in the uterine cavity volume and the extension of the duration of pregnancy preservation after multiple pregnancies are likely to cause the fetus to be overweight and suffer from fetal macrosomia [16, 19]. As the number of pregnancies increases, the weight of the fetus also increases, along with the risk of macrosomia [1, 16, 20–22]. The study by Akin Usta et al. showed a 64% multiparity rate among mothers with fetal macrosomia, as well as a significantly higher parity in the macrosomia group than in the control group [23]. However, related studies on the incidence of fetal macrosomia focused on high parity (more than four pregnancies). The impact of one and two fetuses on the incidence of fetal macrosomia has not been fully confirmed in China, especially after the implementation of the universal two-child policy. Therefore, the current research on fetal macrosomia is necessary. We aim to investigate parturients in the Western China cities of Lanzhou and Chongqing via a cross-sectional study to explore the differences in the incidence of fetal macrosomia in first and second pregnancies. We also put forward potential suggestions and policy recommendations to provide a basis for reducing the incidence of fetal macrosomia.

## Methods

### Study population and methods

This study is part of the social science planning project of Chongqing, China, and includes data from the maternal investigation. This cross-sectional study was conducted from August 2017 to January 2018 in three hospitals (two from Chongqing and one from Lanzhou, Gansu). The subject mothers were surveyed 2–3 days after childbirth in the obstetrics and gynecology departments of each hospital. This study used convenience sampling to naturally divide the subjects into the first and second parturient groups. Finally, 1598 pregnant women were included in the study, including 1094 primiparas and 504 women giving birth to their second child. We also examined the participants of the second pregnancy group and further divided it into the fetal and non-fetal macrosomia groups to identify any potential relationships between macrosomia cases in the second child and those in the first child. The inclusion criteria for parturients were a gestational age of more than 37 gestational weeks and successful single births as first and second births. The exclusion criteria are maternal births with third parity and above; complications in pregnancy and childbirth history, such as therioma, pregnancy associated with cardiac disease, and gynecological diseases; and maternal delivery with congenital malformations. The criteria were implemented to exclude potential confounding and bias effects, such as prematurity and diseases in infants.

### Questionnaire

#### Questionnaire design

The questionnaire was designed by the Public Nutrition and Health Research group, School of Public Health, Medical University of Chongqing. It was optimized according to the relevant literature and revised according to the pre-survey results. The final version of the survey was constructed after multiple discussions by epidemiologists, statisticians, a nutrition professor, and a health management professor from the Medical University of Chongqing. The questionnaire was self-filled and included the pre-investigation and formal investigation.

#### Questionnaire content

The questionnaire consisted of three parts, namely, demographic characteristics, status of health behavior, and pregnancy outcomes. The items on demographic characteristics included ethnicity (Han/minority), only child (Yes/No), height and weight, area (Gansu/Chongqing), residence (town/rural), educational level (primary/intermediate/senior), marital status (married/unmarried/divorced or widowed), per capita income of the family (< 4500¥/4500¥ to 9000¥/> 9000¥), and family history (yes, which/no/unknown). The status of health behavior included the daily physical activity time (< 30 min/> 30 min). The pregnancy outcomes included parity (pregnant women in their first pregnancy/pregnant women in their second pregnancy), gender of the newborn (male/female), maternal BMI(kg/m^2^), neonatal weight, fetal macrosomia (yes/no), mode of delivery (normal childbirth/uterine-incision delivery), and pregnancy-related complications (placenta previa/uterine scarring/pregnancy-induced hypertension/cholestasis of pregnancy/premature rupture of membranes/hydramnion/hypamnion/fetal growth restriction/anemia/thrombocytopenia/pregnancy associated with cardiac disease/pregnancy combined with thyroid disease/pregnancy with viral hepatitis/AIDS/others/nothing) [24].

#### Statistical analyses

Frequencies and percentages values were recorded to describe the demographic characteristics of the first and second pregnancy groups and the second pregnancy group. The mean, standard deviation, Pearson’s chi-square test, and *t* test described the differences in parity and the influential factors of macrosomia in the first and second parturient groups and in the second pregnancy group. Logistic regression analysis was used to explore the differences in the incidence of fetal macrosomia in first and second pregnancies after the adjustment of related variables. These variables are factors that have been identified in previous studies to influence macrosomia incidence, including maternal age (years, < 30/≥30), maternal height (m), delivery weight (kg), maternal BMI (kg/m^2^, < 30/≥30), gestational diabetes mellitus (Yes/No), gestational hypertension (Yes/No), physical activity time (daily) (< 30 min/≥ 30min), and fetal gender (Male/Female) [16–18]. The multivariate model was statistically significant (coefficient test *p* < 0.05) and reached a good fit in the Hosmer and Lemeshow test (*p* = 0.755). Statistical significance was considered at *p* < 0.05. All survey data were encoded using Epidata 3.0. Data analyses were carried out using the statistical software Statistical Package for Social Science version 22 (IBM; Armonk, NY, USA).

## Results

### Distribution of characteristics among participants

A total of 1598 pregnant women were included. Table 1 shows the demographic characteristics of the first and second pregnancy groups. These groups included 1094 (68.5%) primiparas and 504 (31.5%) women giving birth to their second child. The number of participants who are a single child is lower in the second pregnancy group (20.4%) than in the first pregnancy group (32.4%). The first pregnancy group (80.1%) reported higher education levels than the second pregnancy group (73.2%) did.

**Table 1:** Distribution of characteristics among participants

Variables	Pregnant women in their first pregnancy (*n*, %)	Pregnant women in their second pregnancy (*n*, %)	*χ*^2^/*t*	*p* value
Nationality				
Han	1020 (93.2)	464 (92.1)		
Minority	74 (6.8)	40 (7.9)	0.716	0.398
Maternal age (years)			2.207	0.027*
Single-child				
Yes	354 (32.4)	103 (20.4)		
No	740 (67.6)	401 (79.6)	24.016	0.000***
Marital status				
Unmarried	9 (0.8)	3 (0.6)		
Married	1078 (98.5)	498 (98.8)		
Divorced or widowhood	7 (0.7)	3 (0.6)		
Education level				
Basic	116 (10.6)	81 (16.1)		
Secondary	102 (9.3)	54 (10.7)		
Higher	876 (80.1)	369 (73.2)	11.136	0.004**
Location				
Lanzhou	863 (78.9)	389 (77.2)		
Chongqing	231 (21.1)	115 (22.8)	0.589	0.443
Residence				
Urban	951 (86.9)	444 (88.1)		
Rural	143 (13.1)	60 (11.9)	0.623	0.515
The per capita income of the family				
< 4500¥	450 (41.1)	208 (41.2)		
4500¥ to 9000¥	506 (46.3)	214 (42.5)		
> 9000¥	133 (12.6)	82 (16.3)	5.424	0.066

*p < 0.05; **p < 0.01; ***p < 0.0001

### Univariate analysis of the first and second pregnancy groups

Table 2 shows the differences in the incidence of macrosomia and macrosomia-related indicators between the first and second pregnancy groups. These variables are factors that have been identified to influence macrosomia incidence in previous studies. We observed no statistical significance in the incidence of fetal macrosomia in the first (7.2%) and second (7.1%) pregnancy groups. The delivery weight, maternal BMI, and fetal birth weight of the second pregnancy group (62.12 kg, 23.98 kg/m^2^, 3448.73 g) were higher than those of the first pregnancy group (60.68 kg, 23.31 kg/m^2^, 3334.63 g). Moreover, the maternal age of the second pregnancy group (28.80 years) was lower than that of the first pregnancy group (29.21 years).

**Table 2:** Univariate analysis of influential factors of macrosomia in the first and second parturient groups

Variables	Pregnant women in their first pregnancy (*n*, %, mean ± SD)	Pregnant women in their second pregnancy (*n*, %, mean ± SD)	*χ*^2^/*t*	*p* value
Fetal macrosomia				
Yes	79 (7.2)	36(7.1)		
No	1015 (92.8)	468 (92.9)	0.003	0.955
Maternal age (years)	29.21±3.51	28.80±3.39	2.207	0.027*
Maternal height (m)	1.61±0.05	1.61±0.05	1.362	0.174
Delivery weight (kg)	60.68±8.72	62.12±8.38	− 3.109	0.002**
Maternal BMI (kg/m^2^)	23.31±3.21	23.98±3.08	− 3.871	0.000***
Gestational diabetes mellitus				
Yes	39 (3.6)	18 (3.6)		
No	1055 (96.4)	486 (96.4)	0.000	0.995
Gestational hypertension				
Yes	39 (3.6)	33 (6.5)		
No	1055 (96.4)	471 (93.5)	7.134	0.008
Physical activity time (daily)				
< 30 min	873 (79.8)	409 (81.2)		
≥ 30 min	221 (20.2)	95 (18.8)	0.397	0.528
Fetal birth weight (g)	3334.63±620.82	3448.73±758.73	− 3.174	0.002**
Fetal gender				
Male	587 (53.7)	268 (53.2)		
Female	507 (46.3)	236 (46.8)	0.032	0.858

*p < 0.05; **p < 0.01;***p < 0.0001. *SD* standard deviation, *BMI* body mass index

Maternal BMI refers to the last weight recorded before delivery

### Univariate analysis of second-time pregnant mothers

Table 3 shows the difference in first pregnant macrosomia incidence and macrosomia influence factors of the second-time pregnant mothers. The case of mothers who have given birth to a second child with macrosomia (5.5%) was not related to whether the first child had macrosomia (4.7%). The mothers in the non-fetal macrosomia group (28.88 years) were older than those in the fetal macrosomia group (27.75 years), and they (19.7%) were trend to be more engage in physical activity than those in the fetal macrosomia group (5.5%).

**Table 3:** Univariate analysis of factors that influence macrosomia in second-time pregnant mothers

Variables	The second child is fetal macrosomia (*n*, %, mean ± SD)	The second child is non-fetal macrosomia (*n*, %, mean ± SD)	*χ*^2^/*t*	*p* value
The first child is fetal macrosomia				
Yes	2 (5.5)	22 (4.7)		
No	34 (94.5)	446 (95.3)	0.000	0.999
Maternal age (years)	27.75±2.39	28.88±3.45	− 2.620	0.012*
Maternal height (m)	161.61±5.00	160.92±4.85	0.819	0.413
Delivery weight (kg)	63.92±8.01	61.99±8.41	1.332	0.184
Maternal BMI (kg/m^2^)	24.47±2.95	23.94±3.09	1.006	0.315
Gestational diabetes mellitus				
Yes	2 (5.5)	16 (3.4)		
No	34 (94.5)	452 (96.6)	0.040	0.842
Gestational hypertension				
Yes	2 (5.5)	20 (4.3)		
No	34 (94.5)	448 (95.7)	0.000	0.999
Physical activity time (daily)				
< 30 min	34 (94.5)	376 (80.3)		
≥ 30 min	2 (5.5)	92 (19.7)	4.382	0.036*
Fetal gender				
Male	20 (55.6)	249 (53.2)		
Female	16 (44.4)	219 (46.8)	0.074	0.785

*p < 0.05. *SD* standard deviation, *BMI* body mass index

Maternal BMI refers to the last weight recorded before delivery

### Multivariate logistic regression analysis

Table 4 shows the multivariate logistic regression model of the differences in the incidence of fetal macrosomia in the first and second pregnancies after adjusting for potentially related confounding variables. As shown in Table 4, parity (first and second pregnancies) has no effect on the occurrence of macrosomia after adjusting the related variables. Mothers older than 30 years are not likely to give birth to infants with macrosomia (OR 0.6, 95% CI 0.4, 0.9)

**Table 4:** Bivariate logistic regression analysis of factors that affect delivery involving fetal macrosomia

Variables		*B*	*χ*^2^	*p* value	OR (95% Cl)
Parity					
Pregnant women in their first pregnancy	1094 (68.5)				1
Pregnant women in their second pregnancy	504 (31.5)	− 0.048	0.053	0.818	0.9 (0.6,1.4)
Maternal age					
< 30	939 (58.8)				1
≥ 30	659 (41.2)	− 0.515	5.963	0.015*	0.6 (0.4,0.9)
Maternal height					
< 1.60	495 (31.0)				
≥ 1.60	1103 (69.0)				
Maternal BMI					
< 30	1547 (96.8)				1
≥ 30	51 (3.2)	0.747	3.029	0.082	2.1 (0.9, 4.9)
Gestational diabetes mellitus					
Yes	57 (3.6)	0.180	0.138	0.710	1.2 (0.5, 3.1)
No	1541 (96.4)				1
Gestational hypertension					
Yes	72 (4.5)	0.582	2.376	0.123	1.8 (0.9, 3.8)
No	1526 (95.5)				1
Physical activity time (daily)					
< 30 min	1282 (80.2)	0.449	2.585	0.108	1.6 (0.9, 2.7)
≥ 30 min	316 (19.8)				1
Fetal gender					
Male	855 (53.5)				1
Female	743 (46.5)	− 0.028	0.020	0.888	1.0 (0.7, 1.4)

*p < 0.05. *BMI* body mass index, *OR* odds ratio, *CI* confidence interval

Maternal BMI refers to the last weight recorded before delivery

## Discussion

With the improvement of the living standards of Chinese people, the incidence of fetal macrosomia has increased [11–15]. The increase in maternal parity after the implementation of the universal two-child policy has resulted in the relaxation of the peritoneum and uterine wall, an increase in uterine volume, and an extension of the duration of pregnancy preservation after multiple pregnancies; these conditions lead to an increased risk of macrosomia [1, 16, 19–22]. We assume that macrosomia incidence in second children will increase. However, our study showed that no statistical significance was found in the incidence of fetal macrosomia in the first and second pregnancy groups. Additionally, the birth of a second child with macrosomia was not significantly related to the occurrence of macrosomia in the first child. However, this finding is not without practical significance. Some studies have shown that increased parity is associated with a high risk of fetal macrosomia [21, 22]. In a study from Sack, mothers with multiple pregnancies had a higher risk of having fetal macrosomia than those in the control group did [22]. Dor et al. reported that the multiparity rate was approximately 70% in the case group [21]. Similarly, the study of Akin Usta et al. showed a multiparity rate of 64% among mothers with fetal macrosomia and a significantly higher parity in the macrosomia group than in the control group [23]. Results from the aforementioned studies are based on mothers with more than two pregnancies. By contrast, the current study focused on only the first and second pregnancies. Therefore, potentially removing the effects of peritoneal and uterine walls and the uterine cavity volume of pregnant women on giving birth to a second child is possible. In the context of China’s “two-child policy,” we have strong evidence that suggests that Chinese mothers may not have to be concerned with the possibility of having a second child with macrosomia. The prevention of fetal macrosomia may primarily start with lifestyle intervention.

This study found that mothers older than 30 years are not likely to give birth to babies with macrosomia. This result varies from foreign research. Some foreign studies discovered that mothers aged 30 years may be at risk for macrosomia [16]. A population in the UK showed that the incidence of macrosomia in women between 35 and 39 years increased by 40% relative to women under 35 years old [25]. Our research also showed that the compared with those in the second pregnancy group, the mothers in the first pregnancy group were older and received higher education. We speculate that this result may be due to the fact that relative to pregnant women under the age of 30, pregnant women over the age of 30 in China are more educated and have better cognition of maternal and child health care. Studies also showed that mothers with higher education have a lower risk of macrosomia than mothers without higher education have [26]. However, mothers in their second pregnancy may possibly have extensive knowledge or education about pregnancy-related health. Education has been recognized as the most important social factor affecting the health of mothers and children [27]. Health and perinatal-educated mothers can maintain a healthy lifestyle, which is important for avoiding poor perinatal outcomes [26]. Previous studies showed that the risk factors for perinatal outcomes such as macrosomia are clearly related to perinatal education [28, 29]. Although the first pregnancy group reported higher education levels, the second pregnancy group had more perinatal experience, which may be the reason why no significant difference was found in the macrosomia incidence between the first and second pregnancy groups. In the context of China’s universal two-child policy, we believe that community and health departments should strengthen perinatal health education, which potentially plays an important role in preventing macrosomia and other adverse pregnancy outcomes. In addition, gestational diabetes and gestational hypertension during pregnancy are very important risk factors affecting the output of huge babies [16–18]. However, because this is a cross-sectional study, it is impossible to know exactly when the pregnant woman suffered from gestational diabetes and/or hypertension in pregnancy, and it is uncertain whether she has been treated at the survey site during her illness, so that it cannot be proved. The causal relationship between the output of giant children has certain limitations. This suggests that in the future we can establish a cohort study to further explore the relationship between the disease and the output of macrosomia in combination with clinical biochemical indicators and public health nutritional indicators, so as to observe that pregnant women are affected by gestational diabetes and/or gestational hypertension, and the connection between control and huge output.

We found that the occurrence of macrosomia in the second child was not significantly affected by whether the first child was born with macrosomia. This finding may indicate that for two pregnancies of the same woman, the birth of a second child with macrosomia is not related to whether the first child had macrosomia. The duration of daily physical activity of the non-fetal macrosomia group was greater than that of the macrosomia group. This result is consistent with those of previous studies. The American College of Obstetricians and Gynecologists, the Royal College of Obstetricians and Gynaecologists, and the Royal College of Midwives collectively recommend 30 min of daily moderate-intensity physical activity for low-risk pregnant women regardless of the stage of pregnancy [30–32]. Regular physical activity can lower the risk of gestational diabetes and thus reduce the macrosomia incidence [31, 33, 34]. However, many Caucasian women with higher education and income and those who plan to not have other children in the family think that activities may be unsafe, exhausting, and uncomfortable, hence their inactivity [35, 36]. In sum, reasonable and regular physical exercise during pregnancy can effectively reduce the occurrence of macrosomia.

### Limitations of the study

The limitations of the study include the following. First, we chose only two regions of Western China to conduct surveys, and thus, our work cannot fully reflect the reality of China as a whole. Second, our data were collected within 5 months, and seasonal variations could lead to time bias. Third, in response to the hospital’s request, we conducted a questionnaire survey using a registered form. The respondents could have been worried about revealing personal information and thus concealed facts, which could cause information bias. Finally, taking into account the small sample size of this survey and the limited population of the survey, it may be possible to expand the sample size in future studies to verify the clear relationship between effect indicators and outcome indicators. All these potential limitations should be considered when the results are interpreted.

## Conclusions

The incidence of macrosomia in Western China might not be affected by second childbirth and is not increased by low parity.

## Data Availability

The datasets used and analyzed during the current study are available from the corresponding author on reasonable request.

## Abbreviations

BMI: Body mass index
SD: Standard deviation
OR: Odds ratio
CI: Confidence interval
AIDS: Acquired immune deficiency syndrome
